# Vaspin accelerates the proliferation, invasion and metastasis of Triple‐Negative breast cancer through MiR‐33a‐5p/ABHD2

**DOI:** 10.1002/cam4.5241

**Published:** 2022-09-20

**Authors:** Xin‐Hui Cao, Xiu Chen, Kai Yang, Ya‐Lin Wang, Ming‐Xing Liang, Yin‐Jiao Fei, Jin‐Hai Tang

**Affiliations:** ^1^ School of Clinical Medicine Xuzhou Medical University Xuzhou People's Republic of China; ^2^ Department of General Surgery The First Affiliated Hospital of Nanjing Medical University Nanjing People's Republic of China

**Keywords:** ABHD2, miR‐33a‐5p, obesity, triple‐negative breast cancer, vaspin

## Abstract

**Objective:**

To explore the influence and the underlying mechanism of vaspin (visceral adipose tissue‐derived serpin) on the development of triple‐negative breast malignancy.

**Methods:**

First, we analyzed medical records and screened out 22 breast cancer patients with different BMI according to inclusion and exclusion criterion, and measured serum vaspin of those patients. Then we studied the effects of vaspin on TNBC cell lines by using EdU assay, colony formation, transwell and wound‐healing assay. Later, we used bioinformatics analysis to identify downstream effectors and verify with qRT‐PCR, luciferase assay, western blot, etc.

**Results:**

We found the vaspin level was positively correlated with BMI in breast malignant patients and vaspin could significantly enhance the proliferation, infiltration and transferring of triple‐negative breast cancer cells by restraining the expression of miR‐33a‐5p. By using bioinformatic analysis and luciferase assay, we identified miR‐33a‐5p directly regulating ABHD2.

**Conclusion:**

Vaspin, as a cancer‐promoting cytokine, may inhibit miR‐33a‐5p thus increasing the level of ABHD2 to promote the development of the triple‐negative breast cancer.

## Graphical abstract

We found that compared with normal‐weight breast cancer patients, serum vaspin levels in overweight patients were significantly increased, and vaspin could promote the progression of triple‐negative breast cancer by regulating the miR‐33a‐5p/ABHD2 pathway

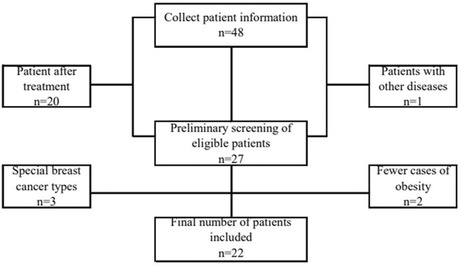

## 1 INTRODUCTION

Breast cancer is one of the upmost causes of female cancer deaths worldwide.^1^, ^2^ According to latest study, the incidence of breast cancer accounted for 19.2% of female cancers in China in 2018, and the mortality rate of breast cancer patients was about 9.1%.^3^ Clinical case analysis showed that obesity could be a risk factor for awful prognosis among breast cancer patients.^4^, ^5^ Among all subtypes of breast cancer, triple‐negative breast cancer (TNBC) is the most aggressive subtype with poor prognosis. Analysis of different subtypes of obese breast cancer patients showed that obese patients with TNBC would develop into a worse prognosis.^6^

Adipokines are components of the cancer cells associated microenvironment and play important roles in the occurrence and development of cancer cells.^7^ In 2005, researchers discovered a novel type of adipose factors called vaspin, which was derived from visceral fat and belonged to the serine protein inhibitor family.^8^ Human serum vaspin protein consists of 415 amino groups and has 40.5% identity with α_1_‐antitrypsin.^8^ A variety of tissues in the human body produced vaspin, like adipose tissue, liver, pancreas, skin, skeletal muscle and adipose tissue, and among them, the production of vaspin in the liver is the highest.^9^, ^10^ The study has found that vaspin concentrations in serum are positively correlated with Body Mass Index (BMI), waist circumference, and body fat percentage.^11^ Meanwhile, other study also showed that vaspin levels could be significantly increased in obese subjects.^12^ Previous study showed the elevated serum vaspin level in patients with endometrial cancer and colorectal cancer was associated with the malignant degree.^13^, ^14^ Regarding breast cancer, so far there are few studies about the influences of vaspin upon breast cancer.

MicroRNAs (miRNAs) belong to the class of non‐coding RNA molecules that possess approximately endogenous 22 bases. Literatures had already reported the diverse features of dysregulated miRNAs in progression of varieties of cancers including breast, stomach, lung and prostate cancer.^15^ It has been determined that miRNAs related to cell behavior, such as proliferation, inflammation, stress response, migration, invasion, differentiation, and apoptosis.^15^, ^16^ Interestingly, microRNA‐33a‐5p (miR‐33a‐5p) was confirmed to be down‐regulated in melanoma as well as hepatocellular carcinoma, representing its suppression on cancer progression.^17^, ^18^ However, the role of miR‐33a‐5p in breast tumor progression holds obscure.

We recently found vaspin could inhibit the expression of miR‐33a‐5p in TNBC cell lines and identified a latent target of miR‐33a‐5p called a/b‐hydrolase domain containing 2 (ABHD2). Previous studies showed that in ovarian cancer ABHD2 lead to a series of adverse reactions like anoikis resistance, chemoresistance and so on. For prostate cancer, ABHD2 promoted cell proliferation and migration. ABHD2 was related to modulating Akt/p70S6K and JNK, thus promoting the malignant progression of cells.^19^ Thus, we hypothesized that vaspin could promote the progress of the TNBC by inhibiting miR‐33a‐5p thus inducing ABHD2 expression.

In the present study, we showed that vaspin was associated with poor prognosis of TNBC patients. Additionally, we found vaspin promoted the proliferation, invasion, and metastasis of triple‐negative breast cancer by suppressing the expression of miR‐33a‐5p. Furthermore, we proved that ABHD2 was a direct target of miR‐33a‐5p in TNBC and miR‐33a‐5p inhibited cell proliferation, migration and invasion in TNBC cells by regulating ABHD2 expression. Therefore, our study reveals vaspin as a novel cancer‐promoting cytokine through miR‐33a‐5p.

## 2 MATERIALS AND METHODS

### 2.1 Patients and samples

**FIGURE 1:**
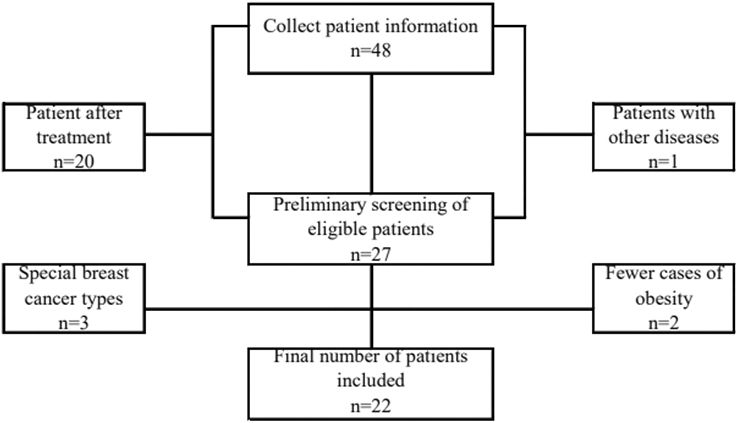
Flow chart of inclusion and exclusion of serum vaspin levels in breast cancer patients.

After screening, 22 Asian patients with invasive breast cancer diagnosed in the Department of Breast Surgery of Jiangsu Provincial People's Hospital were included in the study (Figure 1). The selective criteria were as follows, inclusion criteria: (1) age: from 18 to 75 year‐old; (2) gender: female; (3) never received surgery, chemotherapy, radiotherapy or other anti‐tumor treatment previously; (4) the breast cancer diagnosis was confirmed by needle biopsy or surgical pathology; (5) all participants voluntarily signed an informed consent before sample collections; (6) the size of the mass was greater than or equal to 150 mg. Exclusion criteria: (1) Complicated with basic diseases such as hypertension and diabetes; (2) Complicated with infection or other inflammatory reactions; (3) Combined with other primary tumors, such as gastric cancer, colorectal cancer, ovarian cancer; (4) Those who have used other drugs in clinical trials within 4 weeks before the first medication; (5) Subjects have congenital or acquired immune deficiencies (such as autoimmune hepatitis, interstitial pneumonia, uveitis, enteritis).

Then we used test tubes free of pyrogen and endotoxin to collect blood from patients as required. After centrifugation at 3000 rpm for 10 min, the serum was quickly and carefully stored at −80°C. Next, we measured the vaspin in the serum according to the instructions of the human vaspin ELISA test kit (Xinquan, China). Based on the BMI, patients were divided into normal (BMI < 24) and overweight (BMI:24–28) groups, with 11 patients in each group.

### 2.2 Cells and cell culture

Human breast cancer cell lines MDA‐MB‐231 were purchased from the Cell Bank of the Chinese Academy of Sciences (Shanghai, China) and Human breast cancer cell lines SUM1315MO2 were sponsored by Stephen Ethier (University of Michigan, Ann Arbor, MI, USA). The culture condition were Dulbecco's modified Eagle's medium (DMEM) with high glucose (HyClone), 10% fetal bovine serum (FBS), and 1%–1.5% penicillin with streptomycin (Cellbio). And the atmosphere of culture were humidity with 5% CO2 at 37°C.

### 2.3 Real‐time quantitative PCR (RT‐qPCR)

To evaluate the optimal concentration and culture time of vaspin affecting the level of miR‐33a‐5p, as well as the transfection efficiency of miRNA mimics, we extracted the total RNAs with TRIzol reagent on the basis of manufacturer's instructions and converted them into complementary DNAs through HisScript II Q RT SuperMix for qPCR (Vazyme). The procedure of PCR was preformed following the instructions of ChamQ SYBR qPCR Master Mix (High ROX Premixed) (Vazyme) on the StepOne Plus Real‐Time PCR System (Thermo Fisher Scientific, USA). The relative expression of miR‐33a‐5p was calculated by the 2^−ΔΔCt^ method, and the internal reference was U6. Human miR‐33a‐5p reverse transcription primer:5′‐GTCGTATCCAGTGCAGGGTCCGAGGTATTCGCACTGGATACGACTGCAAT‐3′ Forward Primer:5′‐CG CGGTGCATTGTAGTTGC‐3′ Reverse Primer:5′‐AGTGC AGGGTCCGAGGTATT‐3′.

In the same way, we measure the downstream genes of miR‐33a‐5p, and the internal reference was GAPDH. The primer sequences are as follows: ABHD2: Forward Primer:5′‐CATGCTGGAGACTCCCGAAC‐3′ Reverse Primers:5′‐CAAACACCGGACGATCACGTA‐3′; ARID5B:Forward Primer:5′‐TCTTAAAGGCAGACCACGCAA‐3′ Reverse Primers:5′‐TGCCATCGGAATTGTTGTTGG‐3′; ABCA1:Forward Primer:5′‐ACCCACCCTATGAACAACA TGA‐3′ Reverse Primers:5′‐GAGTCGGGTAACGGAAAC AGG‐3′; IRS2:Forward Primer:5′‐CGGTGAGTTCTACGG GTACAT‐3′ Reverse Primers:5′‐TCAGGGTGTATTCATCC AGCG‐3′. All the above primers are synthesized by Sangon Biotech Co. Ltd.

### 2.4 Transfection of miR‐33a‐5p

Human miR‐33a‐5p mimics were purchased from RiboBio Company (Guangzhou, China). According to the protocol, miR‐33a‐5p mimics were transfected into MDA‐MB‐231 and SUM1315MO2 cells (5 × 10^5^ cells/well) through an usage of Lipofectamine™ 3000 transfection reagent (Invitrogen). After 8 h, cells were continued to culture for 48 h with the replaced medium containing 10% FBS.

### 2.5 Western blot assay

Cells were rinsed with cold PBS and with RIPA buffer (Beyotime) on ice for half an hour. The lysate was centrifuged at 12,000 g, 4°C for 20 min. BCA protein determination kit (Beyotime) was employed to measure the protein concentration. Then the protein was separated by SDS‐PAGE and transferred to a polyvinylidene fluoride (PVDF) membrane, which was blocked with 5% skimmed milk powder in TBST (Tris 0.05% Tween‐20), incubated with primary antibody against ABHD2 (1:1000 dilution. Proteintech) and GAPDH (1:10000 dilution. Proteintech) at 4°C overnight, washed with TBST, and incubated with HRP‐conjugated secondary antibody (1:1000 dilution. Proteintech) for 1 h at room temperature. Then bands were visualized with an enhanced chemiluminescence (Beyotime) system.

### 2.6 Cell migration and invasion assay

By the transwell experiment, the functions of vaspin and miR‐33a‐5p upon the migration and invasion of MDA‐MB‐231 and SUM1315MO2 cells were determined. For transwell migration assay, MDA‐MB‐231 and SUM1315MO2 cells were firstly treated with vaspin or transfected with miR‐33a‐5p mimics. After 48 h, cells were digested with trypsin, resuspended in a serum‐free medium and placed in the upper layer of the chamber (1x10^4^ cells/well). Completed medium containing 20% serum were added to the lower chamber. The chamber was fixed with 4% paraformaldehyde (Servicebio)and dyed with 1% crystal violet (Sangon biotech) 24 h later. The cells inside the upper layer of the chamber were gently cleaned by a cotton swab. Then the cells migrated at the bottom of the chamber were imaged. For the invasion experiment, first of all, the chamber was covered with diluted Matrigel. The subsequent procedures were in accordance with the migration experiment. Because SUM1315MO2 has a weaker invasion ability, the incubation time is 48 h, while MDA‐MB‐231 is still 24 h.

### 2.7 Wound healing

First, the treated MDA‐MB‐231 or SUM1315MO2 cells in 6‐well plates were cultured to a confluency of 90%–95%. Then we used a sterile 200 μL pipette to scrape a linear wound across the cell layer. Floated cells and debris were washed by PBS and adherent cells were cultured in serum‐free medium. Finally, the width of the wounds was pictured via the microscope camera (Canon, Japan) instantly. Because of the differences in cell growth rate and migration ability between, the width of the wounds of MDA‐MB‐231 cells were taken after 24 h, and the SUM1315MO2 cells were taken after 48 h. The ratio of (1‐existent area/initial area) × 100% represents the migration ability of cells.

### 2.8 Cell proliferation assay

The colony formation experiment and the EdU assay (RiboBio) were performed to evaluate the proliferation ability of cells. MDA‐MB‐231 and SUM1315MO2 cells were treated with vaspin or transfected with miR‐33a‐5p mimics. After 48 h, cells were digested with trypsin and inoculated into a 6‐well plate (800 cells/well) for the colony formation experiment or into 96‐well plates (10^4^ cells/well) for the EdU assay. For the colony formation experiment, the cells were fixed and stained with 4% paraformaldehyde and 1% crystal violet, respectively, after 10 days of culture. Then we observed and counted the generated cell colonies. For the Edu cell proliferation assay, after 24 h of growth, the cells were operated in terms of the instructions of the Edu cell proliferation kit. The picture was obtained by using an inverted fluorescence microscope and the data were analyzed.

### 2.9 ABHD2 3'UTR construct and luciferase reporter gene detection

Design PCR amplification primers for ABHD2‐WT or ABHD2‐MUT, PCR amplify the 3'UTR sequence of the gene, purify and recover the target fragment, connect it to a dual fluorescent reporter vector, transform DH5α competent *E. coli*, spread the plate, pick a single clone for colony PCR identification, and finally DNA sequencing verified the constructed plasmid vector (RiboBio). Using Lipofectamine 3000 (Invitrogen), MDA‐MB‐231 or SUM1315MO2 cells were co‐transfected with ABHD2‐WT or ABHD2‐MUT and miR‐33a‐5p or miR‐NC. After 48 h, the luciferase activity was evaluated by the dual‐luciferase reporter system (Vazyme) in accordance with the manufacturer's protocol.

### 2.10 Bioinformatics and statistical analysis

Target genes of miR‐33a‐5p were predicted by miRDB (http://mirdb.org/), TargetScan (http://www.targetscan.org/), and miRTarBase (http://mirtarbase.cuhk.edu.cn/php/index.php). GEPIA database (http://gepia.cancer‐pku.cn/) was used to analyze difference in the gene expression of breast cancer. Kaplan–Meier Plotter website (http://www.kmplot.com/breast) was utilized to analyze the survival correlation between ABHD2 expression and breast cancer. The results were displayed as the mean ± SD. All experiments were independently repeated three times. GraphPad Prism version 8.0.2 software was used for data analysis. Differences between two groups were calculated by Student's *t*‐test. Differences among three or more groups were performed with the one‐way analysis of variance. *p* < 0.05 was regarded as statistically significant.

## 3 RESULTS

### 3.1 Serum vaspin level was positively correlated with BMI among breast cancer patients

**FIGURE 2:**
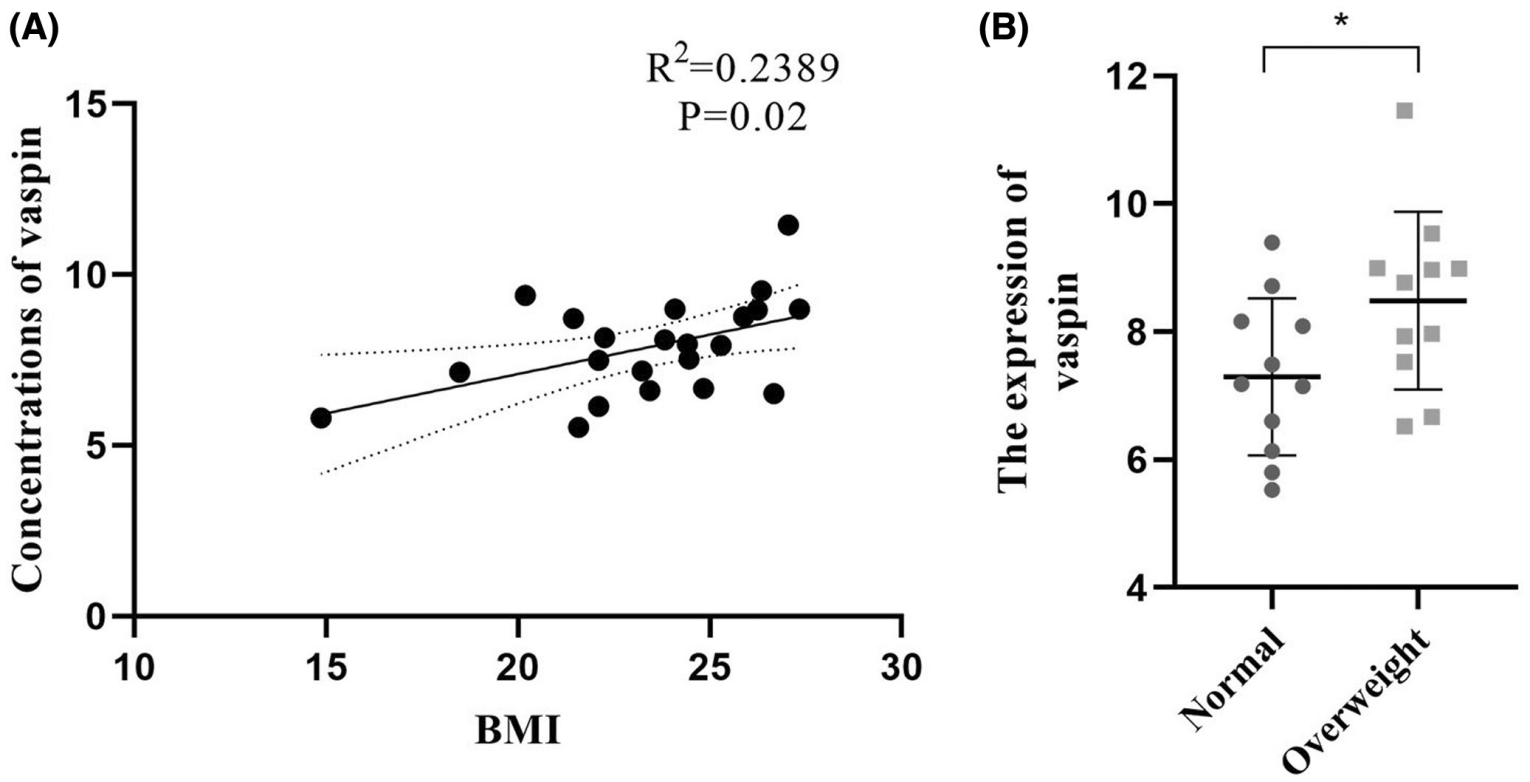
The relationship between serum vaspin content and BIM index. (A). The difference in vaspin content between normal body reconstitution and super recombination, (normal *n* = 11, overweight *n* = 11), *p < 0.05; (B). The linear relationship between serum vaspin content and BMI (*R* ^2^ = 0.2389, *p* = 0.02).

Table 1 showed the clinicopathology information of 22 patients included in the following analysis. We first analyzed vaspin level in patients'serum and found that the average vaspin content in the serum of the overweight group was higher than that of the normal one with statistical significance (Figure 2A). We further analyzed whether there was a linear relationship between vaspin and BMI. As shown in Figure 2B, the result came that a certainly positive linear relationship between serum vaspin content and BMI was emerged.

**TABLE 1:** Clinicopathology information of breast cancer patient

Characteristics	Case No	Percentage
Age (years)		
> = 75	0	0%
<75	22	100%
Gender		
Male	0	0%
Female	22	100%
BMI		
<24	11	50%
24–28	11	50%
> = 28	0	0%
ER		
Positive	15	68.18%
Negative	7	31.82%
PR		
Positive	11	50%
Negative	11	50%
Her‐2		
Positive	5	22.73%
Negative	17	77.27%
KI67		
> = 14%	4	18.18%
<14%	18	81.82%
Blood sugar		
High	0	0%
Normal	22	100%
Low	0	0%
Blood pressure		
High	0	0%
Normal	22	100%
Low	0	0%
Other illnesses		
Yes	0	0%
No	22	100%
Whether to treat		
Yes	0	0%
No	22	100%

### 3.2 Effect of vaspin on the expression of miR‐33a‐5p in breast cancer cells

**FIGURE 3:**
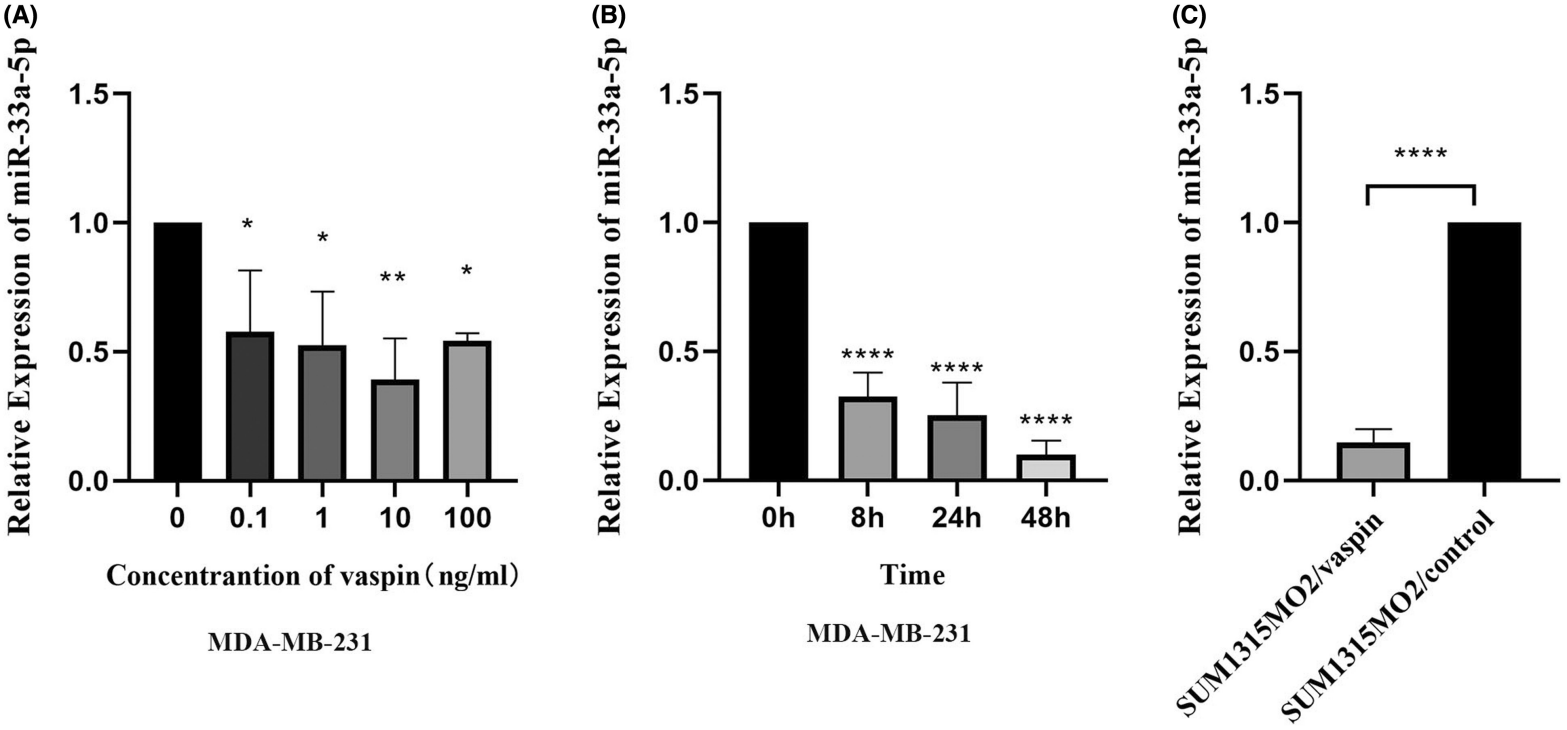
Vaspin inhibited the expression of miR‐33a‐5p in breast cancer cells. (A, B) MDA‐MB‐231 cells were cultured by medium added multipy concentrations of vaspin (0, 0.1, 1.0, 10, 100 ng/mL) for 24 h or 10 ng/mL vaspin for different time periods (0, 8, 24, 48 h) culture, measure the expression of miR‐33a‐5p; (C) The different expression of miR‐33a‐5p SUM1315MO2 cells interfered with 0 or 10 ng/mL vaspin for 48 h. All data were the mean ± SD of three independent experiments, and each performed in triplicate. *p < 0.05, **p < 0.01, ***p < 0.001, ****p < 0.0001 versus 0 ng/mL or 0 h.

Previous studies showed vaspin inhibits miR‐33a‐5p in THP‐1 macrophages‐derived foam cells. To reveal the influence of vaspin on miR‐33a‐5p expression in breast cancer cells, we treated MDA‐MB‐231 cell line with 0, 0.1, 1, 10, 100 ng/mL concentration gradient for 24 h, and then the expression of miR‐33a‐5p was measured by qPCR. As demonstrated in Figure 3A, vaspin decreased the expression of miR‐33a‐5p in MDA‐MB‐231 cell, with largest inhibitory effect at 10 ng/mL. Next, we treated the MDA‐MB‐231 cell line with 10 ng/mL vaspin for 0, 8, 24, and 48 h. As shown in Figure 3B, 48 h treatment of vaspin had largest inhibitory effect on miR‐33a‐5p expression. To further confirm the inhibitiory influence of vaspin on miR‐33a‐5p, we treated SUM1315MO2 with 10 ng/mL for 48 h and used qPCR to evaluate miR‐33a‐5p level. As exhibited in Figure 3C, vaspin treatment significantly decreased the miR‐33a‐5p expression in SUM1315MO2 cells.

### 3.3 Vaspin promotes proliferation, invasion, and metastasis of TNBC cells

**FIGURE 4:**
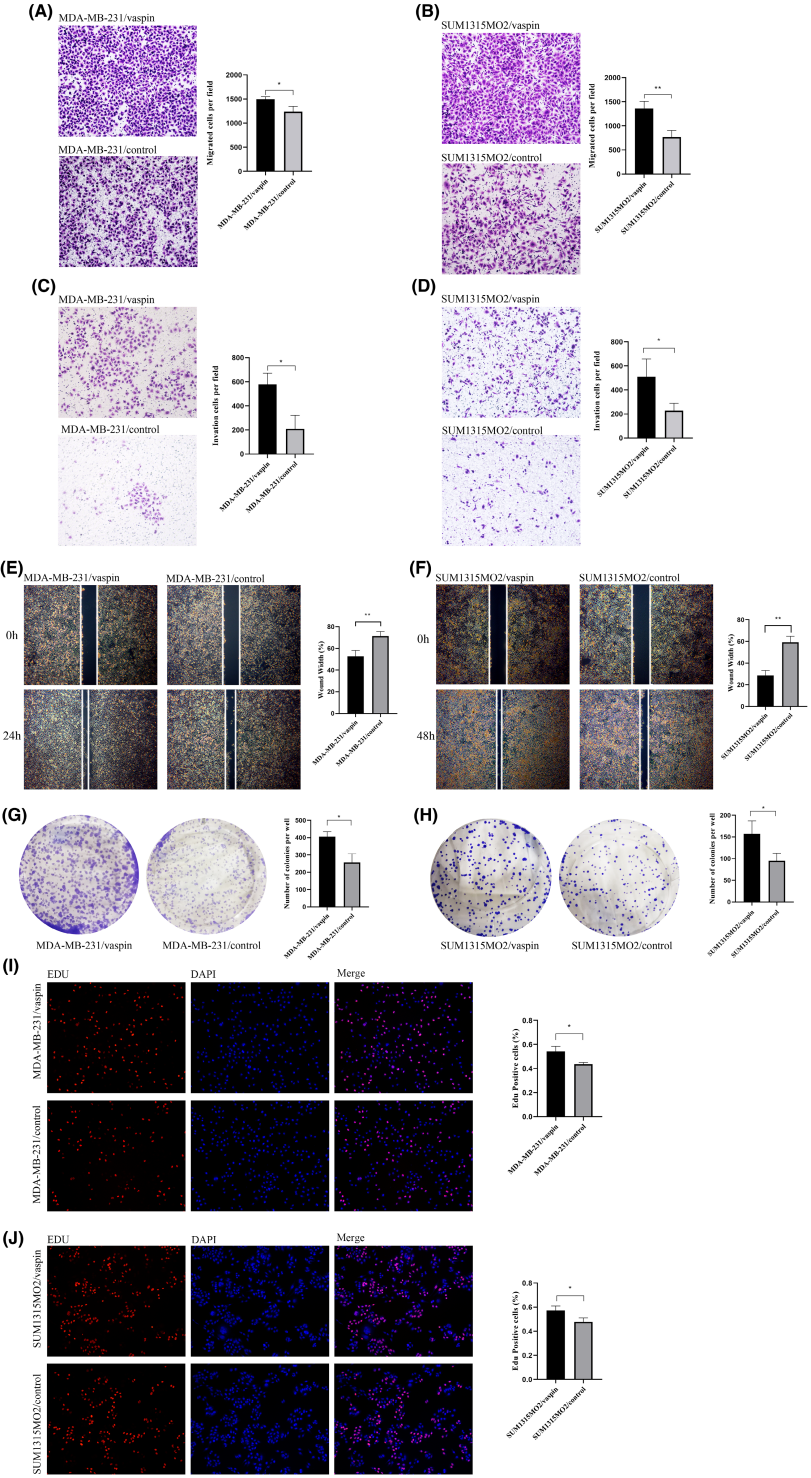
Vaspin promoted migration, invasion and metastasis of triple‐negative breast cancer cells. (A, B) The 24 h Transwell migration test results and analysis of MDA‐MB‐231 and SUM1315MO2 cells interference group and control group (100 × magnification); (C)The 24 h Matrigel invasion test results and analysis of MDA‐MB‐231 cells interference group and control group (100 × magnification); (D) The 48 h Transwell invasion test results and analysis of SUM1315MO2 cells a interference group and control group (100 × magnification); (E)The 24 h wound‐healing experiment results and analysis of MDA‐MB‐231 cells interference group and control group (40 × magnification); (F)The 48 h wound‐healing experiment results and analysis of SUM1315MO2 cells interference group and control group (40 × magnification); (G, H) The 14d Colony formation experiment results and analysis of MDA‐MB‐231 and SUM1315MO2 cells interference group and control group; (I, J) The 24 h EdU cell proliferation assay results and analysis of MDA‐MB‐231 and SUM1315MO2 cells interference group and control group (100 × magnification); All data were calculated by the mean ± SD of three independent experiments. *p < 0.05, **p < 0.01 versus the control.

We next studied the functional role of vaspin on TNBC cell. We treated the MDA‐MB‐231 and SUM1315MO2 cells with 10 ng/mL vaspin for 2 days. The differences in migration and invasion ability between the treated group and the untreated group were observed through transwell and wound healing experiments. The results were shown in Figure 4A–F, vaspin could significantly promote the cell migration and invasion. Figure 4G–J verified the effect of vaspin on the cell proliferation via the Edu and colony formation assays. Results showed that vaspin might enhance the evolution of TNBC cells.

### 3.4 miR‐33a‐5p inhibited the proliferation, invasion, and metastasis of TNBC cell lines

**FIGURE 5:**
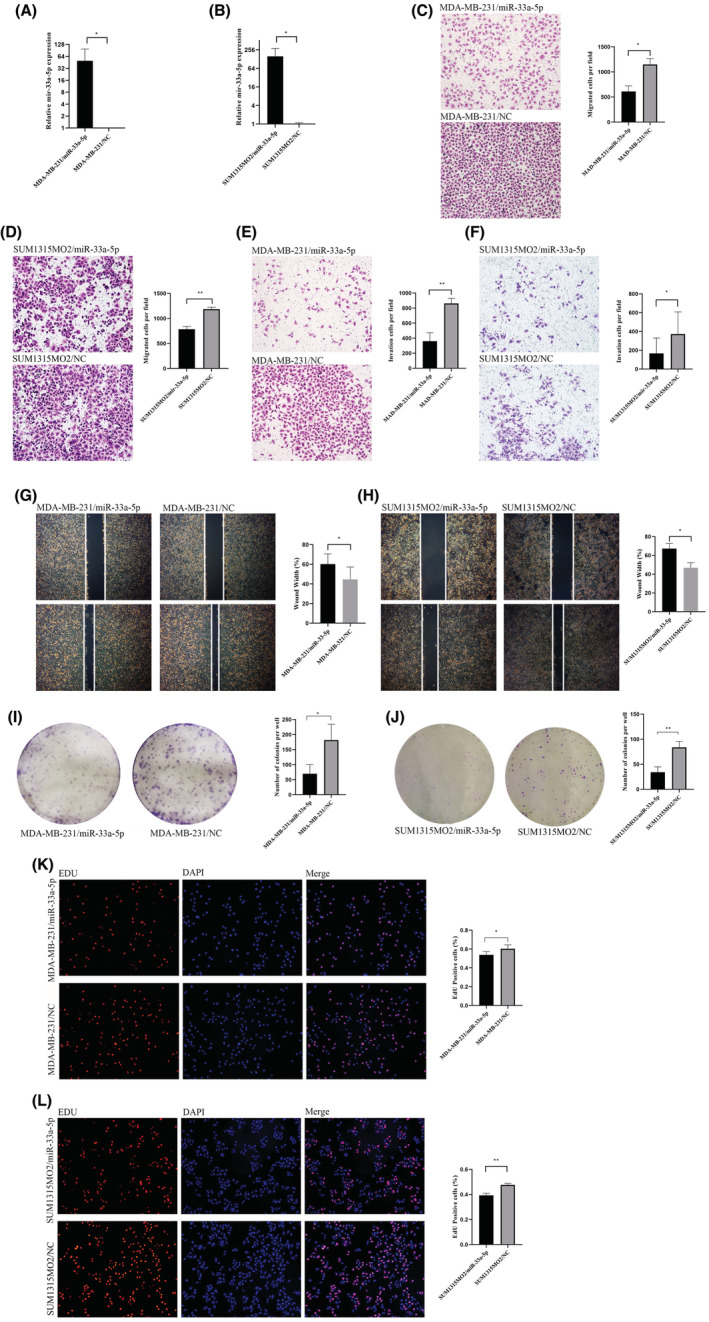
miR‐33a‐5p inhibited migration, invasion and metastasis of triple‐negative breast cancer cells. (A, B) RT‐qPCR results demonstrated that miR‐33a‐5p expression were obviously elevated in MDA‐MB‐231 and SUM1315MO2 cells transfected with miR‐33a‐5p mimic in comparison with the control groups.; (C, D) The 24 h Transwell migration test results and analysis of MDA‐MB‐231 and SUM1315MO2 miR‐33a‐5p‐mimic group and NC group (100 × magnification); (E) The 24 h Transwell invasion test results and analysis of MDA‐MB‐231 experimental group and NC group (100 × magnification); (F) The 48 h Transwell invasion test results and analysis of SUM1315MO2 experimental and NC group (100 × magnification); (G)The 24 h wound‐healing experiment results and analysis of MDA‐MB‐231 cells experimental and NC group (40 × magnification); (H)The 48 h wound‐healing experiment results and analysis of SUM1315MO2 cells experimental and NC group (40 × magnification); (I, J) The 14d Colony formation experiment results and analysis of MDA‐MB‐231 and SUM1315MO2 cells experimental and NC group; (K, L) The 24 h EdU cell proliferation assay results and analysis of MDA‐MB‐231 and SUM1315MO2 cells experimental and NC group (100 × magnification); All data were showed as the mean ± SD and triplicates were performed. *p < 0.05, **p < 0.01 versus NC group.

Previous studies have shown that vaspin could reduce the expression of miR‐33a‐5p. Here we validated the influence of miR‐33a‐5p TNBC cell lines. Both MDA‐MB‐231 and SUM1315MO2 cell lines were transfected with miR‐33a‐5p mimics and scrabble mimics. And 48 h after transfection, the miR‐33a‐5p level were significantly increased in both cell lines (Figure 5A,B). Migration assay and wound healing assay showed overexpression of miR‐33a‐5p could make the TNBC cells migration ability decline (Figure 5C,D, G,H). Invasion assay showed overexpression of miR‐33a‐5p weakened invasion ability in TNBC cell line (Figure 5E,F). Both colony formation assay and EdU assay showed that TNBC cells proliferation ability could be promoted by overexpression of miR‐33a‐5p (Figure 5I–L). These results confirmed the proliferation, invasion, and migration abilities of TNBC cell could be inhibited by miR‐33a‐5p.

### 3.5 miR‐33a‐5p reverses the effect of vaspin on TNBC

**FIGURE 6:**
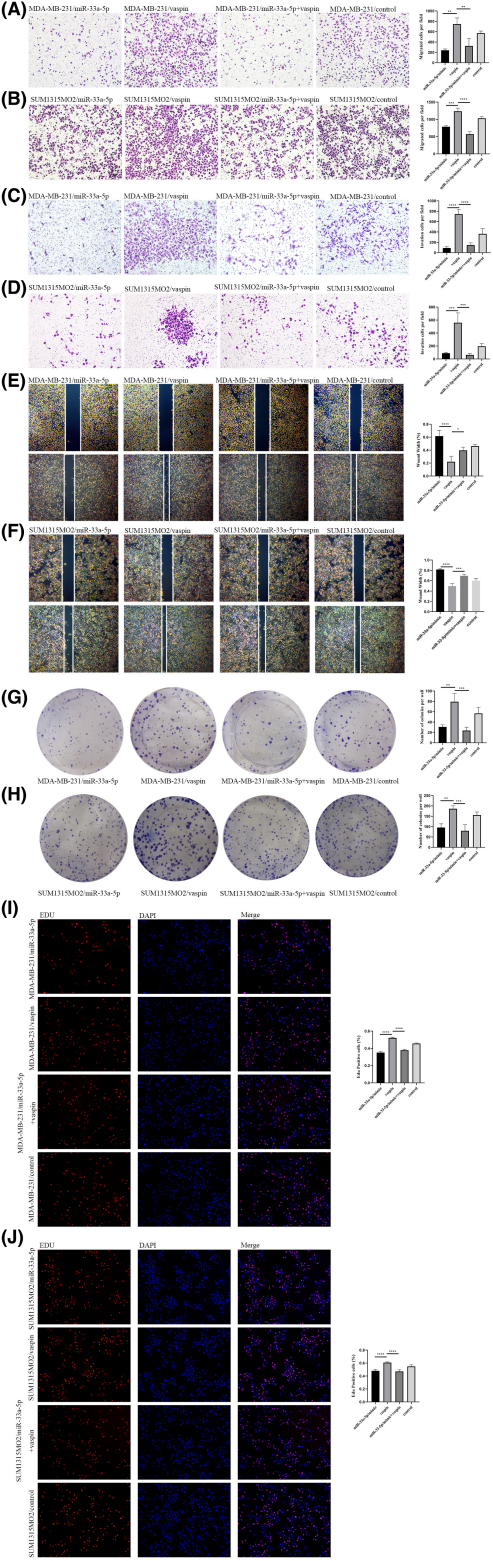
miR‐33a‐5p reversed the effect of vaspin on triple‐negative breast cancer (A–J) MDA‐MB‐231 cells and SUM1315MO2 cells were given transfection miR‐33a‐5p, vaspin interference, transfection miR‐33a‐5p and vaspin interference, or no operation. Consistent with the previous conditions, the cells in the above treatment groups were subjected to transwell migration experiment, transwell invasion experiment, wound‐healing experiment, cell colony formation experiment and EdU cell proliferation assay. At the same time, an analysis of differences between treatment groups was performed. All experiments were performed in triplicates. *p < 0.05, **p < 0.01.

To further seek whether miR‐33a‐5p can inhibit the promotion of vaspin on the invasion, invasion, metastasis and proliferation of MAD‐MB‐231 and SUM1315MO2 cells, miR‐33a‐5p mimic, vaspin, vapsin+miR‐33a‐5p mimic and blank groups were subjected to phenotypic experiments. Results of the transwell experiment discovered that the cell number in the vapsin+miR‐33a‐5p group was remarkably less than that in the vaspin group, indicating that miR‐33a‐5p might abate the ability of vaspin to promote cell migration and infiltration (Figure 6A–D). Wound‐healing experiments presented that in comparison to the vaspin group, the wound healing speed of vapsin+miR‐33a‐5p was significantly slower, declaring that miR‐33a‐5p would eliminate the metastasis‐promoting energy of vaspin (Figure 6E,F). Similarly, in the colony generation experiment (Figure 6G,H) and EdU (Figure 6I,J) experiment, we found that the number of colonies and the proportion of proliferating cells produced by the miR‐33a‐5p group and the vapsin+miR‐33a‐5p group were similar. While, compared to the vaspin group, cells in the vapsin+miR‐33a‐5p group were significantly reduced. Therefore, miR‐33a‐5p might reverse the proliferative action of vaspin in the triple‐negative breast cancer cells.

### 3.6 miR‐33a‐5p directly targets ABHD2

**FIGURE 7:**
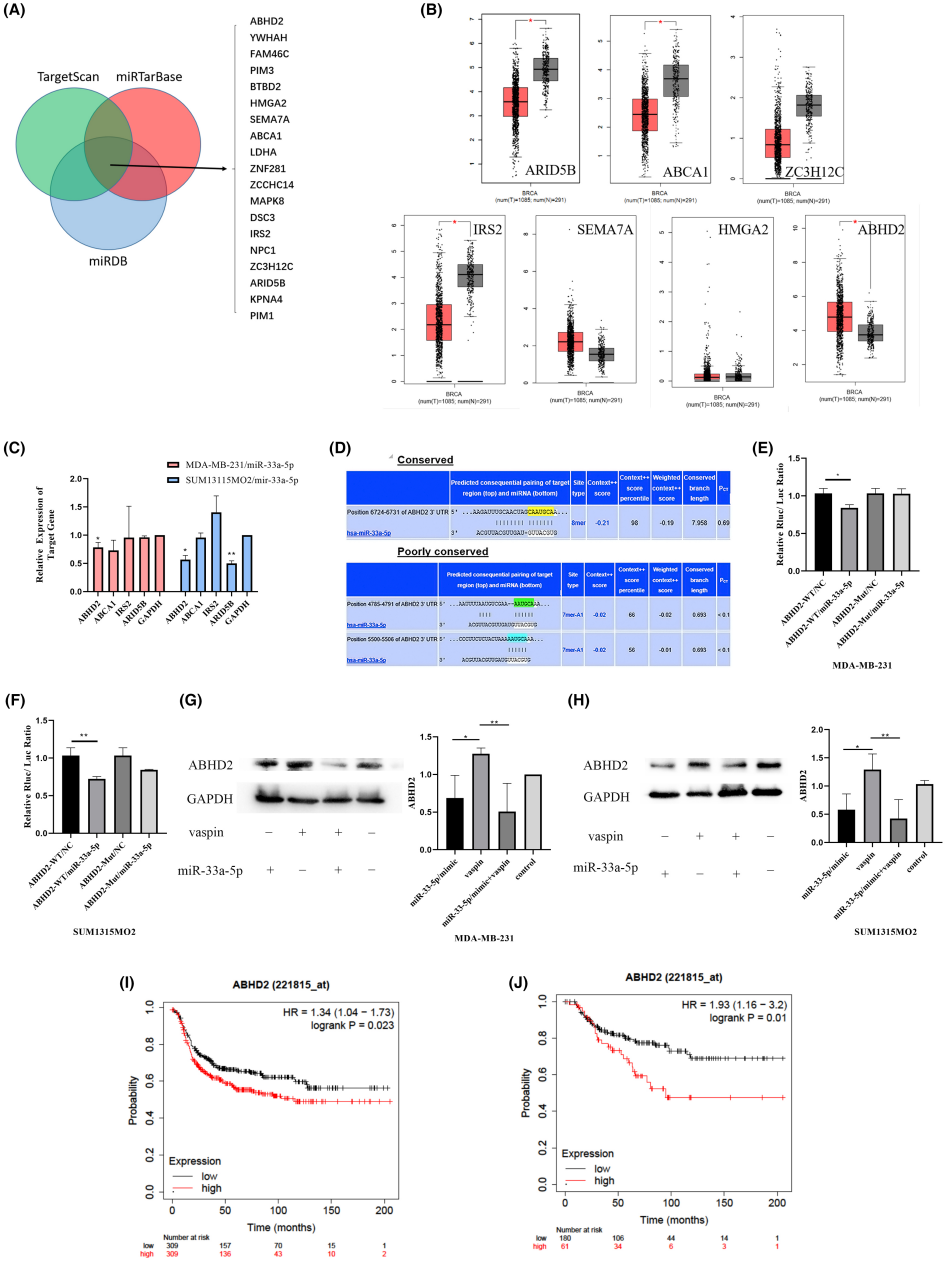
(A) Used Venn diagram to analyze miR‐33a‐5p downstream target genes in miRDB, miRTarBase and TargetScan databases. (B) GEPIA database shows the differential expression ARID5B, ABCA1, ZC3H12C, IRS2, SEMA7A, HMGA2, and ABHD2 in breast cancer. (C) RT‐qPCR results showed differential expression of ABHD2, ABCA1, ARID5B and IRS2 in miR‐33a‐5p/mimic or NC‐transfected MDA‐MB‐231 and SUM1315M02 cells. (D) The TargetScan software predicts that there is a potential binding site for miR‐33a‐5p on the 3'UTR of the ABHD2 gene. (E, F) MDA‐MB‐231 cells and SUM1315MO2 cells were co‐transfected with a wild‐type or mutant 3′‐UTR sequence of ABHD2 and the miR33a‐5p mimic or NC, and the luciferase activity was measured at 48 h and analyzed. (G, H) Western blot experiment to determine the expression and analysis results of the ABHD2 gene in MDA‐MB‐231 cells and SUM1315MO2 cells miR‐33a‐5p/mimic, vaspin, vaspin+miR‐33a‐5p/mimic and the control group. (I) The Kaplan–Meier Plotter database was used to estimate PFS with low (*n* = 309) or high (*n* = 309) levels of ABHD2 in patients with triple‐negative breast cancer. (J) The Kaplan–Meier Plotter database was applied to estimate the OS of TNBC patients with low (*n* = 180) or high (*n* = 61) levels of ABHD2. Experimental data were performed in triplicates. *p<0.05, **p<0.01

We used miRDB (http://mirdb.org), miRTarBase (http://mirtarbase.mbc.nctu.edu.tw/index.html) and TargetScan (http://www.targetscan.org)databases to analyze the downstream genes of miR‐33a‐5p, and intersected the results of the three data. As it showed in Figure 7A, 19 genes showed in all predicted results from three database. As it showed in Figure 7B, ARID5B, ABCA1, IRS2, and ABHD2 were differentially expressed in breast cancer tissues based on GEPIA (http://gepia.cancer‐pku.cn) database among those 19 genes. Then we transfected the miR‐33a‐5p/mimics and NC groups and used qPCR to evaluate mRNA level of those 4 genes. The result showed miR‐33a‐5p could inhibit ABHD2 in both MDA‐MB‐231 and SUM1315MO2 cells with significant differences, while ARID5B was obviously decreased in MDA‐MB‐231 but without significant differences in SUM1315MO2, also other 2 genes were not significantly affected in either cell lines (Figure 7C). Previous studies showed ABHD2 promoted cell proliferation and migration. Here we found ABHD2 was overexpressed in breast cancers. In order to verify that miR‐33a‐5p binds to ABHD2 wild‐type 3'‐UTR or mutant 3'‐UTR, luciferase assay was applied in MDA‐MB‐231 cells and SUM1315MO2 cells (Figure 7D). The luciferase assay showed that the overexpression of miR‐33a‐5p inhibited the luciferase activity of the ABHD2‐MT‐3'‐UTR reporter, but not the ABHD2‐MUT‐3'‐UTR reporter (Figure 7E,F).

Next, we divide the MDA‐MB‐231 and SUM1315MO2 cells into vaspin+miR‐33a‐5p/mimic, vaspin, miR‐33a‐5p/mimic and blank treatments, and then collected the protein to observe the downstream gene ABHD2 gene protein expression level. The results are shown in Figure 7G,H. These results indicated vaspin could induce ABHD2 expression by inhibiting miR‐33a‐5p amplification, and upregulation of miR‐33a‐5p could decrease ABHD2 expression with or without vaspin induction.

Finally, We used the database K‐M Plotter (https://kmplot.com/analysis) to figure up the impact of ABHD2 on the disease‐free survival (DFS) rate and overall survival (OS) rate of breast cancer patients. High expression of ABHD2 is detrimental to DFS and OS of breast cancer patients (Figure 7I–G).

## 4 DISCUSSION

According to recent study, Asians have a higher body fat percentage than Caucasians with the same BMI.^20^ Survey studies have found that the proportion of obese and overweight people in China gradually increases over the age, peaks at middle‐aged and elder groups.^21^ Obesity and overweight not only increases the risk of chronic diseases such as high blood pressure, diabetes, and hyperlipidemia but also affects the incidence and prognosis of cancer.^22^ TNBC is a subtype of breast cancer with awful prognosis.^23^ Clinical studies have proved that TNBC patients with obese and overweight have worse prognosis.^24^ In our study, we put forward the idea that the high expression of vaspin in obese patients affected the miR‐33a‐5p/ABHD2 thus promoting the progression of TNBC.

Previous studies showed that people with high BMI had higher vaspin levels in serum.^12^ In this study, we found that breast cancer patients who were overweight had higher serum vaspin level than patients with normal weight. In addition, we showed that increased vaspin might promote the progression of triple‐negative breast cancer. As serum vaspin level can be managed through intervention in weight control (weight loss surgery or diet control),^25^, ^26^ the weight control may be able to reduce the risk of TNBC progression.

It has been previously delivered that miR‐33a‐5p was a tumor suppressor in several types of cancer. Research by Yili Wang and his colleagues discovered that upregulation of miR‐33a‐5p in vitro could target methylenetetrahydrofolate dehydrogenase 2 (MTHFD2) to hold up the growth and migration of rectal cancer cells.^27^ Zhaohui Gong and his coworkers delivered that the tumor suppressors like miR‐33a‐5p and miR‐128‐3p, in the whole blood of lung cancer patients were highly stable and were supposed to be biomarkers for early lung cancer diagnosis.^28^ At present, there are few studies on miR‐33a‐5p in breast cancer. For example, the study of Ji Wu et al. uncovered that the overexpression of miR‐33a‐5p could significantly improve the sensitivity of TNBC to doxorubicin.^29^ Our study first reported that vaspin could significantly down‐regulate miR‐33a‐5p in TNBC cells, overexpression of miR‐33a‐5p could rescue the cell phenotype change induced by vaspin, indicating vaspin could be used as one of potential upstream targets of miR‐33a‐5p.

ABHD2 is an abhydrolase domain containing 2 which called α/β‐hydrolase containing protein 2. Previous studies have found that ABHD2 played a key role in the infiltration of macrophages into atherosclerosis.^30^ Recent studies have shown that ABHD2 could promote cancer progression. For example, ABHD2 is a direct target of miR‐140‐3p in skin melanoma cells. It could reverse the anti‐tumor effect of miR‐140‐3p, and the influence of miR‐140‐3p on Akt/p70S6K and JNK pathways were reversed by ABHD2 overexpressing.^19^ Currently, there was no report on the role of ABHD2 in breast cancer. Through online database analysis and dual‐luciferase experiments, we supposed that ABHD2 may be a direct target of miR‐33a‐5p in breast cancer cells. Besides, we also proved that the mutual effect between vaspin and miR‐33a‐5p could change the quantity of ABHD2. In this study, we did not focus on downstream signaling of ABHD2. Further studies will be required to reveal the detailed roles and respective mechanisms of ABHD2 in breast cancer.

In conclusion, we found that compared with normal weight breast cancer patients, serum vaspin level in overweight patients significantly increased, and vaspin could promote the progression of TNBC by regulating the miR‐33a‐5p/ABHD2 pathway. Through this study, we could gain an in‐depth understanding of the role of vaspin/miR‐33a‐5p/ABHD2 in obese and overweight TNBC, thus better understanding the mechanism of breast cancer deterioration in obese and overweight people. This study will provide new therapeutic insights for inhibiting the progression of TNBC. In the future study, we will reveal the detailed molecular mechanism of ABHD2 in breast cancer.

## AUTHOR CONTRIBUTIONS

Xinhui Cao and Xiu Chen completed most of the cell experiments and drafted the manuscript. Kai Yang performed clinical case sample collection, measurement, and data analysis. Ya‐Lin Wang and Ming‐Xing Liang complete the data analysis of cell experiments. Yin‐Jiao Fei conducted database data analysis. Jinhai Tang He provided experimental protocol, experimental guidance, and data review.

## FUNDING INFORMATION

Our research was supported by the National Natural Science Foundation of China (No. 81902987 and 81872365), National Key Research and Development Program of China (No. 2016YFC0905900) and Jiangsu Provincial Key Research Development Program (No. BE2019731).

## CONFLICT OF INTEREST

In the future study, we will reveal the detailed molecular mechanism of ABHD2 in breast cancer. All authors declare no conflict of interest.

## ETHICS APPROVAL AND CONSENT TO PARTICIPATE

The experimental protocol was established, according to the ethical guidelines of the Helsinki Declaration and was approved by the Human Ethics Committee of The First Affiliated Hospital of Nanjing Medical University. Written informed consent was obtained from all patients before enrollment.

## Data Availability

The data in this study are open access via https://osf.io/jsnw9/?view_only=12c829a3ac834012b5a82d5a8f0bff05.
